# Effect-Directed Profiling of *Akebia quinata* and *Clitoria ternatea* via High-Performance Thin-Layer Chromatography, Planar Assays and High-Resolution Mass Spectrometry

**DOI:** 10.3390/molecules28072893

**Published:** 2023-03-23

**Authors:** Hanna Nikolaichuk, Irena M. Choma, Gertrud E. Morlock

**Affiliations:** 1 Chair of Food Science, Institute of Nutritional Science, Justus Liebig University Giessen, Heinrich-Buff-Ring 26-32, 35392 Giessen, Germany; 2 Department of Chromatography, Faculty of Chemistry, Maria Curie-Sklodowska University, Maria Curie-Sklodowska Sq. 3, 20031 Lublin, Poland; 3 Department of Bioanalytics, Faculty of Biomedicine, Medical University of Lublin, Jaczewskiego St. 8b, 20090 Lublin, Poland

**Keywords:** HPTLC−EDA, HPTLC–heart cut–HPLC–HESI-HRMS, bioassay, enzyme inhibition assay, inhibitor, genotoxicity, genotoxin, antioxidant, radical scavenger, antibacterial, antimicrobial, estrogen, androgen, agonist, antagonist, endocrine activity

## Abstract

Two herbal plants, *Akebia quinata* D. leaf/fruit and *Clitoria ternatea* L. flower, well-known in traditional medicine systems, were investigated using a non-target effect-directed profiling. High-performance thin-layer chromatography (HPTLC) was combined with 11 different effect-directed assays, including two multiplex bioassays, for assessing their bioactivity. Individual active zones were heart-cut eluted for separation via an orthogonal high-performance liquid chromatography column to heated electrospray ionization high-resolution mass spectrometry (HPLC–HESI-HRMS) for tentative assignment of molecular formulas according to literature data. The obtained effect-directed profiles provided information on 2,2-diphenyl-1-picrylhydrazyl scavenging, antibacterial (against *Bacillus subtilis* and *Aliivibrio fischeri*), enzyme inhibition (tyrosinase, α-amylase, β-glucuronidase, butyrylcholinesterase, and acetylcholinesterase), endocrine (agonists and antagonists), and genotoxic (SOS-Umu-C) activities. The main bioactive compound zones in *A. quinata* leaf were tentatively assigned to be syringin, vanilloloside, salidroside, α-hederin, cuneataside E, botulin, and oleanolic acid, while salidroside and quinatic acids were tentatively identified in the fruit. Taraxerol, kaempherol-3-rutinoside, kaempferol-3-glucoside, quercetin-3-rutinoside, and octadecenoic acid were tentatively found in the *C. ternatea* flower. This straightforward hyphenated technique made it possible to correlate the biological properties of the herbs with possible compounds. The meaningful bioactivity profiles contribute to a better understanding of the effects and to more efficient food control and food safety.

## 1. Introduction

*Akebia quinata* Decaisne and *Clitoria ternatea* Linne are well-known herbs used in various traditional medicine systems, with growing popularity in modern medicine and pharmacy in Europe. Although many Europeans still recognize *A. quinata* as only a garden decoration and *C. ternatea* additionally as a drink (vibrant, blue-colored herbal tea), they have a wide range of pharmacological and biological activities. *Akebia quinata* is a woody climber from the Lardizabalaceae family, commonly known as a chocolate vine. The plant is widespread in East Asia, including Korea, China, and Japan [1,2,3]. In traditional medicines, different plant parts and preparations of *A. quinata* are exploited. Aqueous and alcoholic extracts, which have antioxidant and free radical scavenging properties, were mainly used to treat edema, hypothermia, and rheumatic pain [4,5,6]. Dry ripe fruit and stem extracts were used as anti-inflammatory, sedative, and diuretic agents [7,8,9]. The fruits, leaves, and stems of the *A. quinata* were reported as ingredients of a slimming tea used to treat obesity in traditional Korean medicine [10]. *In vivo* and *in vitro* studies pointed to the antiobesity and hypolipidemic effects of *A. quinata* [11]. Supplementation with *A. quinata* was found to improve induced hyperlipidemia, hyperleptinemia, body weight gain, and adipose tissue weight, without affecting food intake in mice. In an *in vitro* study, it was suggested that the aqueous extract of *A. quinata* had a potential (neuro)therapeutic role in the treatment of stress-induced fatigue [12]. It significantly increased the expression of serotonin, adrenaline, and noradrenaline in the brain and reduced brain atrophy, due to the presence of chlorogenic acid, isochlorogenic acid A, and isochlorogenic acid C. The triterpene glucoside of *A. quinata* stems, Akequintoside F, was reported to have an inhibitory effect on Aβ42 fibrillogenesis [13]. It was suggested that *A. quinata* could be considered a non-toxic source for treating Alzheimer’s disease. The contained saponins demonstrated nitric oxide inhibition and cytotoxicity against cancer cells [14]. Another *in vivo* study [15] pointed to the potential reduction of the ethanol concentration in blood in mice with alcohol-induced hepatotoxicity. This means that *A. quinata* extracts can be used as a competitive hangover beverage, with a beneficial function on hangover relief. The antibacterial activity against *Staphylococcus aureus*, *Bacillus thuringiensis*, *Escherichia coli*, *Salmonella enterica*, and *Shigella dysenteriae* of *Akebia trifoliate* has been extensively studied *in vitro* [16,17], which pointed to the presence of triterpenoids 2a,3b-dihydroxy-23-oxo-olean-12-en-28-oic acid, maslinic acid, arjunolic acid, oleanolic acid, 3-epi-oleanolic acid, and 2a,3b-dihydroxyol-ean-13(18)-en-28-oic acid. Further information on the bioactivity of constituents and their structures is missing for *A. quinata*.

*Clitoria ternatea* L., from the Fabaceae family, also known as the butterfly pea, is a popular plant used in the traditional Ayurvedic medicine. It has been exploited as a brain tonic to treat stress and depression and enhance memory and intelligence [18,19], and it is described to have antioxidant, hypolipidemic, anticancer, anti-inflammatory, analgesic, antipyretic, antidiabetic, antimicrobial, gastro-intestinal antiparasitic, and insecticidal activities, among others [20]. The root powder of *C. ternatea* is used to treat snakebite and scorpion stings in India [21]. An *in vitro* study pointed to the antirheumatic activity of the aqueous root extract of *C. ternatea* [22]. A recent study proved the anti-inflammatory and antirheumatic activity of the *C. ternatea* root extract [23], and another one proved the nootropic and anticholinesterase activities, potentially helpful for cognitive decline treatment [24]. *In vivo* research [25] proved that *C. ternatea* flowers increase the acetylcholine content and acetylcholinesterase activity in rats. Further studies showed the antiasthmatic activity of ethanolic *C. ternatea* root extract [26]. The leaf extract of *C. ternatea* was reported to have antidiabetic, antihyperglycemic, antioxidant [27], neuroprotective, and nootropic activities [28]. The flower extract was proven to have anti-inflammatory and analgesic activity related to the presence of taraxerol [29]. The petal extracts of *C. ternatea* revealed antimicrobial activity, protective effect against hemolysis of erythrocytes, inhibition of lipid peroxidation, reduction of LDL cholesterol and oxidative DNA strand scissions, inhibition of α-amylase, α-glucosidase, and angiotensin-I-converting enzymes, oxygen radical absorption capacity, intracellular antioxidant activity against reactive oxygen species, and no cytotoxicity against the A549, HCT8, and IMR90 cell lines [30]. *C. ternatea* has antioxidant [31,32] and antibacterial properties against both Gram-positive bacteria (*Bacillus cereus*, *B. subtilis*, *B. thuringiensis*, *Staphylococcus aureus*, *Streptococcus faecalis*) and Gram-negative bacteria (*Escherichia coli, Klebsiella pneumoniae*, *Pseudomonas aeruginosa*, *Salmonella typhi*, *Enterobacter aerogens*, *Proteus mirabilis*, *Herbaspirillum* spp.) as well as antifungal activity (*Candida albicans*, *Rhizopus* spp., and *Penicillium* spp.) [33,34,35,36]. Moreover, *C. ternatea* was discussed as a potential drug against the SARS-CoV-2 virus, due to the presence of the flavonoid glucoside kaempferol-3-*O*-α-rhamnopyranosyl(1/2)[α-rhamnopyranosyl(1/6)]-β-glucopyranoside [37].

The identification and determination of chemical constituents of *A. quinata* and *C. ternatea* by various analytical methods have been reported, including HPLC [38,39,40], NMR [41,42,43,44], and mass spectrometry [45,46,47,48]. Although the bioactivity of both plants was studied, a combination of high-performance thin-layer chromatography (HPTLC) separation and effect-directed assay detection on the same adsorbent surface has not been reported. Hence, a non-target HPTLC bioactivity profiling of *A. quinata* and *C. ternatea* was developed. Planar antibacterial, antioxidant, enzyme inhibition, endocrine activity, and genotoxicity (bio)assays were applied to straightforwardly detect the individual bioactive compound zones. These were heart-cut eluted via an orthogonal high-performance liquid chromatography column to the heated electrospray ionization high-resolution mass spectrometry (heart-cut-HPLC–HESI-HRMS) system to tentatively assign molecular formulas.

## 2. Results and Discussion

Three *A. quinata* leaf samples (A1–A3) and one fruit sample (A4) were obtained from botanical gardens, whereas four *C. ternatea* flower products (C1–C4) were bought on the market (Table S1). Each sample was ground, ultrasound-extracted with methanol–water 4:1, which was superior to *n*-hexane or ethyl acetate (Figure S1), and centrifuged. For the development of the non-target, effect-directed profiling method, no target analytes were in mind; thus, the compounds were distributed along the migration distance. HPTLC plates silica gel 60 F_254_ were used, except for the endocrine and genotoxicity bioassays, in which HPTLC plates without F_254_ were required to avoid any interference with the 254 nm detection of the formed fluorescein (end-product of the enzyme–substrate reaction). After studying 16 different mobile phase systems (Table S2), the mixture ethyl acetate–methanol–water−acetic acid 70:15:15:1, *V*/*V*/*V*/*V*, was found suitable. Nevertheless, two adjustments were necessary for three (bio)assays. Due to the complexity of the multiplex planar yeast antagonist-verified estrogen screen and respective androgen screen (pYAVES/pYAVAS) bioassays, the acid was skipped (ethyl acetate−methanol−water 70:15:15, *V*/*V*/*V*) to avoid the plate neutralization step. Due to the apolar assay responses for the α-amylase inhibition assay, the mobile phase system (ethyl acetate–*n*-hexane 1:4, *V*/*V*) was reduced in the solvent strength.

All-in-all, eleven HPTLC chromatograms were prepared and subjected to derivatization via the *p*-anisaldehyde sulphuric acid reagent and to ten different (bio)assays. These were the antioxidant (2,2-diphenyl-1-picrylhydrazyl, DPPH• scavenging) assay, two antibacterial bioassays using Gram-positive *B. subtilis* and Gram-negative *A. fischeri* bacteria, the SOS-Umu-C genotoxicity bioassay, the multiplex endocrine pYAVES/pYAVAS bioassays, as well as five enzyme inhibition assays acting against acetylcholinesterase (AChE), butyrylcholinesterase (BChE), β-glucuronidase, tyrosinase, and α-amylase. The obtained HPTLC profiles were detected at UV/Vis/FLD and, via the instant *A. fischeri* bioluminescence, depicted as a greyscale image. Selected bioactive zones (marked with Roman numerals) were further characterized by heart-cut HPLC–HESI-HRMS.

### 2.1. Effect-Directed Profiling of Akebia quinata

**Figure 1:**
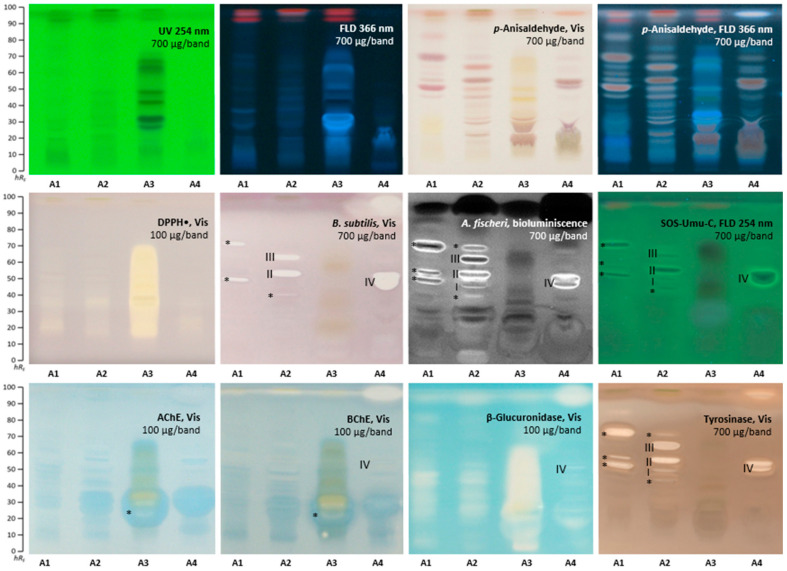
HPTLC profiles of *A. quinata* extracts (A1−A3 leaf and A4 fruit, 100 or 700 µg/band) at UV/Vis/FLD, and after derivatization with the *p*-anisaldehyde sulphuric acid reagent, and after several planar assays (*A. fischeri* bioluminescence depicted as greyscale image), separated on HPTLC plates silica gel 60 F_254_ (without F_254_ for the SOS-Umu-C bioassay) with ethyl acetate–methanol–water–acetic acid (70:15:15:1, *V*/*V*/*V*/*V*).

The HPTLC profiles (Figure 1) detected at UV/Vis/FLD, after derivatization with the *p*-anisaldehyde sulphuric acid reagent and after various (bio)assays showed substantial differences, not only between the three leaf extracts of *A. quinata* (Table S1, A1–A3), but also for the fruit extract (A4). The differences were attributed to various factors affecting the raw material, such as plant part, age, origin, location, and harvest time, to name a few. Already, an applied amount of 100 µg/band exhibited a very strong DPPH• scavenging (antioxidant) activity evident as yellow zones on a purple plate background, especially for the A3 extract (Figure 1). The extracts applied at 700 µg/band showed antibacterial activity against *B. subtilis* and *A. fischeri* bacteria. In contrast to A3, the other leaf extracts A1 and A2 revealed antibacterial activity against *B. subtilis*, observed as colorless zones on a purple plate background. For A1, sharp antibacterial zones were observed at *hR*_F_ 55 and 72 (both marked*), and for A2, at *hR*_F_ 45 (marked*), 60 (zone **II**), and 65 (zone **III**). The fruit extract A4 revealed two strong antibacterial zones at *hR*_F_ 55 (zone **IV**) and *hR*_F_ 97. A comparatively stronger response was observed against *A. fischeri* bacteria, evident as dark or bright zones. Three sharp zones were detected for A1 at *hR*_F_ 55, 60, and 72 (all marked*), while five sharp zones at *hR*_F_ 45 (marked*), 52, 60, 65 (zones **I**–**III**), and 70 (marked*) were evident for A2. Again, in contrast to leaf extracts, a bright zone at *hR*_F_ 55 (**IV**) and a black zone at *hR*_F_ 97 were detected for the A4 fruit extract. The SOS-Umu-C bioautogram revealed all-in-all eight genotoxic zones as bright green fluorescent zones on a green plate background at *hR*_F_ 55, 60, and 72 (all marked*) for A1, *hR*_F_ 45 (marked*), 52, 60, and 65 (zones **I**–**III**) for A2, and *hR*_F_ 55 (zone **IV**) for A4, all applied at 700 µg/band. The sample A3 did not show any genotoxicity. Both the AChE and BChE inhibition assays showed only one inhibition zone at *hR*_F_ 20 (marked*) for A3 (100 µg/band). The fruit sample A4 showed a weak BChE inhibition zone at *hR*_F_ 55 (**IV**) and a strong BChE inhibition zone at *hR*_F_ 97. All samples, especially the leaf extract A3 (100 µg/band), revealed very strong inhibition of the β-glucuronidase visible as a broad colorless area, ranging *hR*_F_ 10–70. The tyrosinase inhibition autogram revealed up to five colorless zones per extract (700 µg/band each), i.e., at *hR*_F_ 55, 60, and 72 (all marked*) for A1, *hR*_F_ 45 (marked*), 52, 60, 65 (zones **I**–**III**), and 70 (marked*) for A2, and *hR*_F_ 55 (zone **IV**) and 97 for A4.

Some zones of *A. quinata* were evident in several (bio)assays, such as the zone at *hR*_F_ 97 for A4 in the antibacterial, as well as BChE, β-glucuronidase, and tyrosinase inhibition assays. Exemplarily, four bioactive zones were chosen for further characterization by HPTLC–HPLC–HESI-HRMS (Table 1). The zones at *hR*_F_ 52, 60, and 65 (**I**–**III**), responsible for antibacterial, genotoxic, and tyrosinase inhibition effects in the A2 leaf extract, were tentatively assigned as syringin, vanilloloside, salidroside, α-hederin, cuneataside E, botulin, and oleanolic acid. The zone at *hR*_F_ 55 (**IV**), responsible for antibacterial, genotoxic, BChE, β-glucuronidase, and tyrosinase inhibition effects in the A4 fruit extract, was tentatively assigned as salidroside and quinatic acid.

The presence of these compounds in *A. quinata* and their pharmacological activities were reported [1,49]. For example, syringin was suggested to have antioxidant, antidiabetic, anti-inflammatory, and antiallergic properties [50], while α-hederin has anticancer potential [51]. Oleanolic acid was described as a compound with antimicrobial, antidiabetic, anti-inflammatory, and antioxidant activity [52]. Concerning betulin, a broad spectrum of biological activities was reported, such as cytotoxicity, as well as anticancer and anti-HIV activity [53]. Quinatic acid showed antibacterial and α-glucosidase inhibition activities, as well as cytotoxicity [54]. The salidroside detected in *A. quinata* leaf and fruit was reported [55] to inhibit the AChE, BChE, α-glucosidase, and tyrosinase, as well as to have free radical scavenging activity. The potential application of salidroside for improving mental performance, as well as preventing and treating ischemic and neurodegenerative diseases, was reported.

### 2.2. Effect-Directed Profiling of Clitoria ternatea

**Figure 2:**
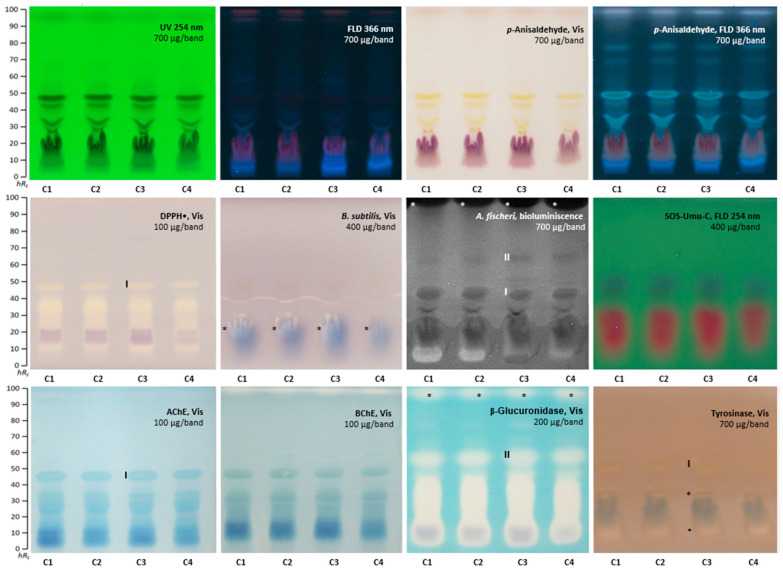
HPTLC profiles of *C. ternatea* flower extracts (C1−C4, 100−700 µg/band) at UV/Vis/FLD, after derivatization with the *p*-anisaldehyde sulphuric acid reagent and after several planar assays (*A. fischeri* bioluminescence depicted as greyscale image), separated on HPTLC plates silica gel 60 F_254_ (without F_254_ for the SOS-Umu-C bioassay) with ethyl acetate–methanol–water–acetic acid (70:15:15:1, *V*/*V*/*V*/*V*).

The four flower extracts of *C. ternatea* (C1-C4, Table S1) were investigated analogously. The antioxidant potential was already observed at an amount of 100 µg/band in all four extracts in the DPPH• bioautogram (Figure 2). One broad antioxidant compound area ranged *hR*_F_ 10−40, and another sharp zone was evident at *hR*_F_ 46 (zone **I**). *C. ternatea* extracts were effective against *B. subtilis* and *A. fischeri* bacteria strains at 400 µg/band and 700 µg/band, respectively. The blue plant pigments coeluted with one antibacterial zone (*B. subtilis* bioautogram, marked*). The previously detected zone **I** (*hR*_F_ 46) was also detected in the *A. fischeri* bioautogram and another zone at *hR*_F_ 56 (zone **II**). However, the strongest response was revealed near the solvent front at *hR*_F_ 98 (marked*), indicating apolar compounds. All four extracts showed no green, fluorescent genotoxic compound zones for the given 400 µg/band applied. A minor AChE and BChE inhibition zone was detected at *hR*_F_ 46 as a colorless zone with a blue halo (zone **I**), and another weak colorless zone near the solvent front was observed in the BChE autogram. Already, at an amount of 200 µg/band, a strong inhibition of the β-glucuronidase was prominent as a colorless area, ranging *hR*_F_ 5−46, and two further zones at *hR*_F_ 56 (zone **II**) and 98 (marked*). Only weak colorless tyrosinase inhibition zones appeared at *hR*_F_ 10, 30 (both marked*), and 46 (zone **I**). The blue sample pigment was evident as a diffused zone in the tyrosinase inhibition bioautogram, partially coeluting with the two weak tyrosinase inhibition zones.

The evaluation of the horizontal *hR*_F_ pattern across the eight different assays pointed to multiple effects arising from the same zone, which can be explained by coelution of various compounds or multipotent activity of one compound. Two active zones were exemplarily chosen for HPTLC–heart-cut−HPLC–HESI-HRMS recording. The zone at *hR*_F_ 46 (**I**) showed antioxidant, antibacterial (against *A. fischeri*), and tyrosinase inhibiting activity, as well as weak AChE and BChE inhibition, and was tentatively assigned to the possibly coeluting taraxerol, kaempherol-3-rutinoside, and quercetin-3-rutinoside (Table 2). The zone at *hR*_F_ 56 (**II)** also showed antibacterial activity against *A. fischeri* and a potent inhibition of the β-glucuronidase, and it was tentatively assigned to the octadecenoic acid and/or kaempferol-3-glucoside.

According to the literature, taraxerol has strong pharmacological potential [56]. Taraxerol has antioxidant, antimicrobial, and antidiabetic properties and is also a potential remedy for neurodegenerative diseases, such as Alzheimer’s. Our findings confirmed the previously reported taraxerol activities. Moreover, it can be concluded that taraxerol has no antibacterial properties against *B. subtilis*, as well as no genotoxic and no β-glucuronidase inhibition activities at the given amounts applied, since zone **I** does not reveal these activities.

### 2.3. Agonistic and Antagonistic Endocrine Profiling of A. quinata and C. ternatea

**Figure 3:**
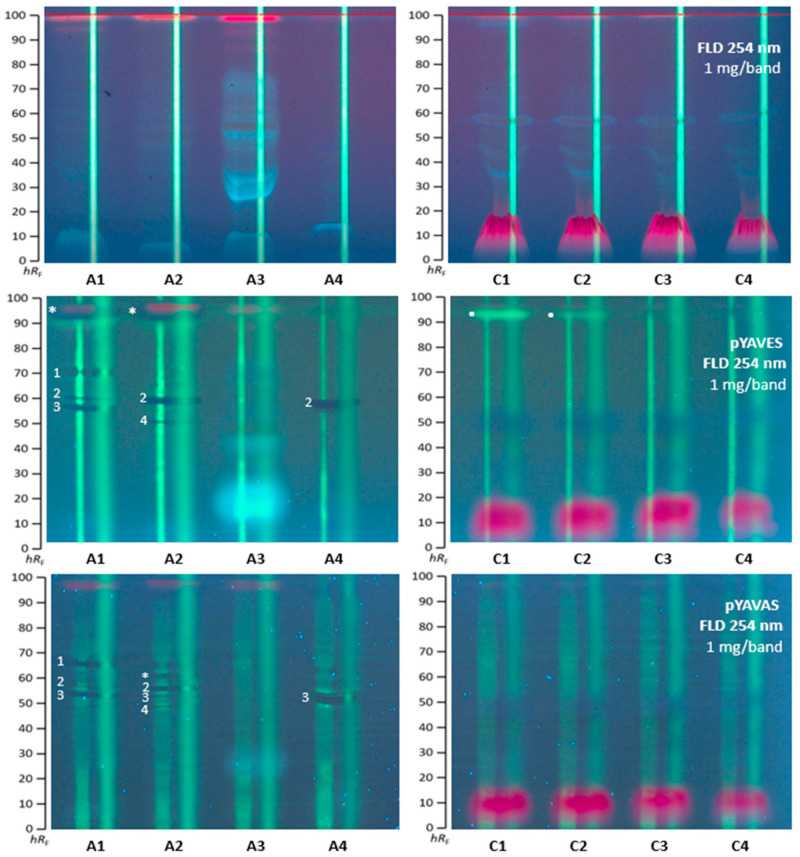
HPTLC profiles at FLD 254 nm before and after the pYAVES/pYAVAS bioassays showing agonistic (^●^) and antagonistic (**1**–**4** and *) endocrine effective compound zones in *A. quinata* and *C. ternatea* extracts (1 mg per 12 mm band, 10 µL/band each) separated on HPTLC plates silica gel 60 using ethyl acetate−methanol−water (70:15:15, *V*/*V*/*V*).

All *A. quinata* and *C. ternatea* extracts were also investigated for agonistic and antagonistic endocrine compounds via the multiplex pYAVES and pYAVAS bioassays. Since the same substrate (as for the genotoxicity bioassay) was used and the same enzyme–substrate end-product, i.e., fluorescein, was formed, HPTLC plates without F_254_ were also used for these multiplex bioassays. Because the acidic mobile phase system required neutralization prior to the bioassay application, three different neutralization buffers were compared (Supplementary Materials Figure S2). However, the acetic acid portion was skipped, since the sharpest zones were obtained in the pYAVAS bioautogram, without any acid and buffering (Figure 3 versus Supplementary Materials Figure S2). The sample volume and bandwidth were increased (1 mg per 12 mm band), as required for the two stripes (1 mm × 70 mm) applied along each separated sample track after the separation. The first agonist stripe (50 pg 17β-estradiol for pYAVES bioassay and 20 ng testosterone for pYAVAS bioassay) was required to detect antagonistic compounds via biologically induced fluorescence reduction of the applied stripe. The second end-product strip (100 ng fluorescein) was needed to detect false-positive antagonists via physico-chemical fluorescence reduction of the applied stripe. Then, the dried chromatogram was treated with a degalan solution for zone fixation, which was required for the multiplex bioassay to keep all responses sharp, but made the layer more apolar. Hence, the chromatogram was subsequently treated with a Tween^®^ 20 solution to allow for a good wettability for the following application of the polar bioassay buffer solutions/suspensions.

*A. quinata* extracts A1, A2, and A4 showed up to four antagonistic compound zones (**1**−**4**) active against estrogens, as well as androgens (Figure 3). On the agonist stripe, the fluorescence-reducing dark bands pointed to anti-estrogenic/-androgenic compounds in the multiplex pYAVES/pYAVAS bioautograms. In the A1 extract, the zones at *hR*_F_ 60, 62, and 70 (**1**–**3**) showed antiestrogenic and antiandrogenic effects, while the zone at *hR*_F_ 97 (marked*) had an antiestrogenic effect, and adjacent below it, a weaker estrogenic response. The A2 extract contained three zones with antiestrogenic activity at *hR*_F_ 52, 62 (**2** and **4**), and 97 (marked*), and four zones with antiandrogenic activity at *hR*_F_ 52, 60, 62 (**2**–**4**), and 67 (marked*). The A3 extract exhibited no activity in both multiplex bioassays. The fruit sample A4 revealed two zones with antiestrogenic activity at *hR*_F_ 62 (**2**) and antiandrogenic activity at *hR*_F_ 60 (**3**). Interestingly, zones **1** and **3** showed a lateral zone/strip focusing property (making the stripe middle more intense), which may indicate a spreading agent property.

The *C. ternatea* samples displayed no antagonistic and androgenic activity. However, a pronounced estrogenic compound zone was detected as a bright green fluorescent zone at *hR*_F_ 98 (marked^●^) in the C1 and C2 extracts. The blue pigment was evident to cause a false-positive antagonistic effect, as evident from the fluorescence reduction on the second stripe.

### 2.4. α-Amylase Effect-Directed Profiling of A. quinata and C. ternatea

**Figure 4:**
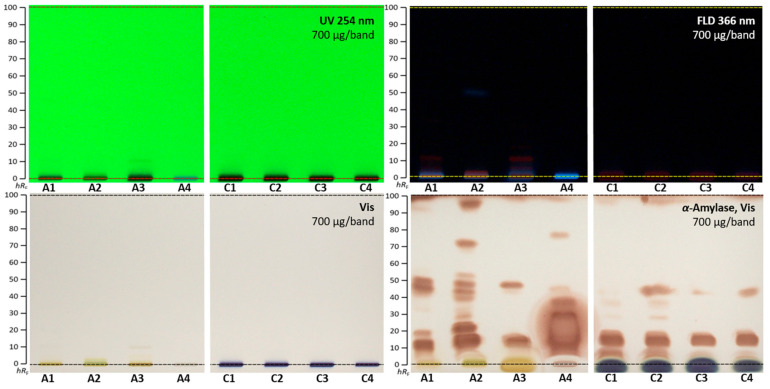
HPTLC profiles of *A. quinata* (A1–A4) and *C. ternatea* (C1–C4) extracts (700 µg/band each) at UV 254 nm, FLD 366 nm, Vis, and after the α-amylase inhibition assay, separated on HPTLC plates silica gel 60 F_254_ using ethyl acetate−*n*-hexane 1:4, *V*/*V*.

The *A. quinata* and *C. ternatea* extracts were studied for their inhibitory activity against the α-amylase. This enzyme degrades polymers into shorter oligomers, participates in carbohydrate metabolism in the human body, and cuts glucose from non-reducing ends of saccharides. Inhibitors of α-amylase limit the digestion and absorption of carbohydrates and, therefore, prevent diabetes, obesity, hyperglycemia, and hyperlipemia [57]. For the separation of α-amylase inhibitors, the given mobile phase system was too strong and, thus, reduced in the solvent strength. The mixture ethyl acetate–*n*-hexane 1:4, *V*/*V*, was found suited. α-Amylase inhibitory activity was observed in all samples applied at 700 µg/band (Figure 4). Up to nine α-amylase inhibiting zones were observed for the *A. quinata* leaf extract A2, while only four were detected for A3. Again, the fruit extract A4 revealed different inhibition zones. All samples of *C. ternatea* fruits had very similar patterns. All detected α-amylase inhibiting compounds are of a more apolar nature and are not detectable at UV/Vis/FLD and, thus, contain no chromophore or fluorophore. These can be of lipidic structure, as recently reported for the α-amylase inhibiting stearic acid and palmitic acid [58].

## 3. Materials and Methods

### 3.1. Chemicals

Bidistilled water was prepared by a Heraeus Destamat Bi-18 E (Thermo Fisher Scientific, Dreieich, Germany). HPTLC plates silica gel 60 F_254_ and HPTLC plates silica gel 60 (both 20 cm × 10 cm) were provided by Merck (Darmstadt, Germany). All chemicals were analytical grade, and all solvents were chromatography grade. Acetylcholinesterase (AChE) from *Electrophorus electricus*, α-amylase from hog pancreas, acarbose, butyrylcholinesterase (BChE) from equine serum, β-glucuronidase from *Escherichia coli*, lysogeny broth powder (containing 5 mg/mL sodium chloride), Gram’s iodine solution, rivastigmine, testosterone, tyrosinase from mushroom, and Tween^®^ 20 were delivered by Sigma-Aldrich (Steinheim, Germany). The (2S)-2-Amino-3-(3,4-dihydroxyphenyl) propionic acid (levodopa) was obtained from Santa Cruz Biotechnology, Dallas, TX, USA. Acetic acid, bovine serum albumin, caffeine, 3-[(3-cholamidopropyl)-dimethylammonio]-1-propanesulfonate (CHAPS), citrate buffer, dimethyl sulfoxide, 2,2-diphenyl-1-picrylhydrazyl (DPPH•), Dulbecco’s phosphate buffered saline (DPBS), ethanol, ethyl acetate, fluorescein di-β-D-galactopyranoside (FDG), gallic acid, glycerol, *n*-hexane, indoxyl acetate, kojic acid, methanol, phosphate buffer, polyethylene glycol (PEG) 8000, D-saccharolactone, tetracycline, thiazol blue tetrazolium bromide (MTT), and tris(hydroxymethyl)aminomethane hydrochloride buffer (TRIS-HCl) were obtained from Carl Roth (Karlsruhe, Germany). The 5-Bromo-4-chloro-3-indonyl-β-D-glucuronide was purchased from Carbosynth (Compton-Berkshire, United Kingdom). The 17β-Estradiol (98.5%) was obtained from Dr. Ehrenstorfer (Augsburg, Germany). The 4-Nitroquinoline-1-oxide (98%) was purchased from TCI (Eschborn, Germany). Degalan^®^ was obtained from Röhm (Darmstadt, Germany). *Aliivibrio fischeri* bacteria (NRRI–B11177, strain 7151) and *Bacillus subtilis* bacteria (DSM-618) were purchased from Leibniz Institute, DSMZ, German Collection of Microorganisms and Cells Cultures (Berlin, Germany). *Salmonella typhimurium* (strain TA1535, genetically modified to contain the plasmid pSK1002) was purchased from Trinova Biochem (Giessen, Germany). *Saccharomyces cerevisiae* BJ1991 containing the human androgen receptor was obtained from Xenometrix (Allschwil, Switzerland). *Saccharomyces cerevisiae* cells equipped with hAR were obtained from Xenometrix, (Allschwil, Switzerland). Additional chemicals and reagents used for cryogenic YAS/YES cell culture have been reported previously [59]. The *A. quinata* samples were obtained from the Botanical Garden, Giessen, Germany (A1 leaf), Palmengarten, Frankfurt, Germany (A2 leaf), and Jagiellonian University, Cracow, Poland (A3 leaf and A4 fruit). The flowers of *C. ternatea* C1−C4 were purchased from vendors in Poland and Germany (Table S1).

### 3.2. Sample Preparation

Each sample was ground (8000 rpm, 5 min, Tube Mill, IKA, Staufen, Germany). At a 1:10 drug−extractant ratio, a 500-mg aliquot was extracted with 5 mL methanol–water 4:1 (*V*/*V*) for 15 min in an ultrasonic bath (20 °C, 100%, 480 W, 35 kHz, Sonorex Digi plus DL 255H, Bandelin, Germany) and centrifuged for 5 min (3000× g, Heraeus Labofuge 400, Thermo Fisher Scientific). The supernatants were stored at −20 °C.

### 3.3. Effect-Directed HPTLC Profiling

The HPTLC plates were prewashed/predeveloped twice with methanol–water 4:1 (*V*/*V*) up to the upper plate edge (Simultan Separating Chamber, biostep, Burkhardtsdorf, Germany), dried in an oven at 110 °C for 20 min, wrapped in aluminum foil, and stored in a desiccator until use. The samples (1−10 µL/band) were applied as 8 mm bands, or 12 mm bands for multiplex bioassays (dosage speed 200 nL/s, first track position 16 mm, distance from the lower plate edge 10 mm and between tracks 16 mm, or 22 mm for multiplex bioassays, Automatic TLC sampler 4, CAMAG, Muttenz, Switzerland). After plate drying (hairdryer, 1 min), the plate was developed up to 70 mm with ethyl acetate−methanol−water−acetic acid 70:15:15:1 (*V*/*V*/*V*/*V*) (if it not stated otherwise) in a Twin Trough Chamber (20 × 10 cm, CAMAG) and dried for 20 min (Automatic Developing Chamber 2, CAMAG). Documentation was performed at 254 nm, 366 nm, or under white light illumination (UV/FLD/Vis, TLC Visualizer, CAMAG). Chromatograms developed with the acidic mobile phase were neutralized with 5% sodium bicarbonate buffer (pH 7.3) via piezoelectrical spraying (yellow nozzle, level 6, Derivatizer, CAMAG) and dried for 10 min. The incubation took place in a polypropylene box (27 cm × 16 cm × 10 cm, KIS, ABM, Wolframs–Eschenbach, Germany) in a humid atmosphere. Each effect-directed assay was performed at least twice, and the reproducibility was confirmed. A respective positive control (PC) [60,61] was applied for each assay.

The DPPH• assay [60] was performed by piezoelectrical spraying 4 mL 0.04% methanolic DPPH• solution (green nozzle, level 4) on the chromatogram. Antioxidants were visible as yellow bands against a purple background at white light illumination. As PC, 0.25 mg/mL gallic acid in methanol (0.2, 0.6, and 1.0 µL/band) was used.

For the *B. subtilis* bioassay [62], the cell suspension (3 mL) was piezoelectrically sprayed on a chromatogram (red nozzle, level 6) and incubated at 37 °C for 2 h. After incubation, MTT solution (0.2% DPBS-buffered) was sprayed on the plate (blue nozzle, level 6), followed by incubation at 37 °C for 30 min. Antibacterials against *B. subtilis* appeared as colorless zones against a purple background at white light illumination. The PC was 0.005 mg/mL tetracycline in ethanol (0.5, 1.5, and 3 µL/band).

The *A. fischeri* bioassay [62] was performed by piezoelectrical spraying 4 mL cell suspension on the chromatogram (red nozzle, level 6). The still humid chromatogram was transferred to the BioLuminizer cabinet (CAMAG). Ten images were recorded over 30 min (exposure time 1 min, trigger interval 3 min). Antibacterials were detected as dark or brightened bands on the instantly bioluminescent plate background (depicted as greyscale image). As PC, 1 mg/mL caffeine in methanol (0.5, 1.5, and 3 μL/band) was used [61].

The SOS-Umu-C bioassay [63,64,65] was performed on HPTLC plates silica gel 60 without F_254_. The genetically modified *S. typhimurium* suspension (2.8 mL) was piezoelectrically sprayed (yellow nozzle, level 3) on the chromatogram, followed by incubation at 37 °C for 3 h. Then, 2.5 mL FDG substrate solution (25 µL of 5 mg/mL FDG in dimethyl sulfoxide and 2.5 mL of phosphate buffer) was sprayed (red nozzle, level 6) on the chromatogram, followed by incubation at 37 °C for 15 min. Genotoxic substances appeared as bright green fluorescent fluorescein bands (released from FDG via the β-galactosidase produced by the bacteria in the presence of DNA-damaging compounds) on a green fluorescent background at FLD 254 nm. The PC was 1 μg/mL 4-nitroquinoline-1-oxide in methanol (1 µL/band).

The AChE/BChE inhibition assays [66] were performed by piezoelectrical spraying (green nozzle, level 5) 1.3 mL substrate solution (1 mg/mL indoxyl acetate in ethanol) on the chromatogram. The plate was dried for 10 min (cold air stream, hairdryer) and sprayed with 3 mL enzyme solution (6.66 U/mL AChE or 3.34 U/mL BChE in Tris-HCl buffer containing 1 mg bovine serum albumin). The incubation at 37 °C took 25 min. White inhibition zones were revealed on the indigo-blue background at white light illumination. As PC, 0.1 mg/mL rivastigmine in methanol (2, 4, and 8 μL/band) was used [61].

The β-glucuronidase inhibition assay [61] was performed by piezoelectrical spraying 2 mL enzyme solution (50 U/mL β-glucuronidase in 0.1 M potassium phosphate buffer, pH 7, containing 1 mg/mL bovine serum albumin) on the chromatogram (yellow nozzle, level 6). After incubation at 37 °C for 15 min, 1.5 mL substrate solution (2 mg/mL aqueous 5-bromo-4-chloro-3-indolyl-β-D-glucuronide solution) was sprayed (yellow nozzle, level 6) on the chromatogram. The incubation at 37 °C took 1 h. β-Glucuronidase inhibition appeared as white bands against an indigo-blue background at white light illumination. As PC, 0.1 mg/mL D-saccharolactone in water (0.8, 1.5, 3 µL/band) was used.

For the tyrosinase inhibition assay [67], 2 mL substrate solution (4.5 mg/mL of levodopa in 20 mM phosphate buffer, pH 6.8, plus 2.5 mg CHAPS and 7.5 mg PEG 8000) was piezoelectrically sprayed (blue nozzle, level 5) on the chromatogram. After drying for 2 min (hairdryer), the chromatogram was sprayed with 2 mL tyrosinase enzyme solution (400 U/mL in phosphate buffer). Incubation was performed in the dark at room temperature for 20 min. Inhibition zones appeared as white bands on a grey background at white light illumination. As PC, 0.1 mg/mL kojic acid in ethanol (1, 3, and 6 μL/band) was used.

The multiplex pYAVES/pYAVAS bioassays were performed as described [59]. The samples were applied (10 μL/band, 12 mm band, 22 mm track distance) on HPTLC plates silica gel 60 without F_254_ and separated with ethyl acetate–methanol–water 70:15:15, *V*/*V*/*V*. pYAVES or pYAVAS bioassays were used to detect both agonistic and antagonistic activity against estrogens and androgens, respectively. Each track on the developed chromatogram was oversprayed with two stripes (1 mm × 70 mm, FreeMode option of winCATS software). For the first stripe (considered as positive control), 4 μL testosterone (5 μg/mL in methanol) was sprayed for the pYAVAS bioassay and 5 μL 17β-estradiol (10 ng/mL in ethanol) for the pYAVES bioassay. The second end-product stripe was 2 μL fluorescein (50 μg/mL in methanol). Then, the chromatogram was immersed for 10 min in a Degalan solution (0.25% in *n*-hexane) and dried for 10 min. Next, the chromatogram was sprayed with 2.5 mL Tween 20 solution (0.02% in ethanol) and dried for 10 min. The chromatogram was sprayed (red nozzle, level 6) with 2.8 mL of the respective cell suspension. The chromatogram was incubated for 4 h (pYAS) or 3 h (pYES) at 30 °C. After incubation, the plate was sprayed (yellow nozzle, level 6) with 2.5 mL FDG solution (as mentioned), followed by incubation at 37 °C for 15 min. Endocrine antagonists reduced the green fluorescence of the testosterone or 17β-estradiol strips, while agonists were detected as bright green fluorescent bands at 254 nm.

The α-amylase inhibition assay was performed, as described recently [61]. The enzyme solution (62.5 U/mL in sodium acetate buffer, pH 7) was sprayed (2 mL, red nozzle, level 6), followed by incubation at 37 °C for 30 min, spraying (1 mL, red nozzle, level 6) with the substrate solution (2% starch in water), another incubation at 37 °C for 20 min, and spraying with Gram’s iodine solution (0.5 mL, yellow nozzle, level 6). As a positive control, acarbose was used (0.1 mg/mL in methanol; 0.3, 0.6, and 0.9 μL/band).

### 3.4. HPTLC–Heart-Cut–HPLC–HESI-HRMS

For HRMS recording, the *A. quinata* A2 and A4 and *C. ternatea* C3 extracts (7 µL/band each) were applied in triplicate on two MS-grade HPTLC F_254_ plates. The active zones of interest were eluted for 1 min with water−methanol (9:1, *V*/*V*) at a flow rate of 0.1 mL/min using the open-source modified auto-TLC-LC-MS interface [68]. The analytes were transferred through a 50-µL sample loop with an integrated desalting cartridge (Accucore RP-MS, 10 mm × 2.1 mm, 2.6 μm, Thermo Fisher Scientific) to the analytical HPLC column (Accucore RP-MS, 100 mm × 2.1 mm, 2.6 μm, Thermo Fisher Scientific) set to 40 °C. Solvent A (2.5 mM ammonium acetate in water, pH 4.5) and solvent B (methanol) were used at a flow rate of 0.4 mL/min for gradient elution, i.e., 0−2 min 2%B, 2−7 min increase to 100%B, hold, and 10−12 min decrease to 2%B. [61] The eluent was directed to the HESI-HRMS system (Q Exactive Plus, Thermo Fisher Scientific). The spectrometer parameters were as follows: capillary temperature 270 °C, spray voltage ± 3.5 kV, sheath gas 20 arbitrary units, aux gas 10 arbitrary units, S–Lens RF level 50. Full scan mass spectra *m*/*z* 100–1100 were recorded in the positive and negative ionization modes. The spectra were processed by Xcalibur 3.0.63 software (Thermo Fisher Scientific).

## 4. Conclusions

Effect-directed profiling via hyphenated HPTLC was found to be a suitable approach for screening the biological activity of *A. quinata* leaf/fruit and *C. ternatea* flower extracts. The meaningful bioactivity profiles extend the presently still limited knowledge on the individual bioactive components of both plants and contribute to a better understanding of their bioactivity potential and to more efficient food control and food safety. The *A. quinata* extracts showed antioxidant, antibacterial (against *B. subtilis* and *A. fischeri*), and enzyme (AChE, BChE, tyrosinase, β-glucuronidase, and α-amylase) inhibition properties. Surprisingly, the extracts also revealed up to four genotoxins. The multiplex pYAVES and pYAVAS bioautograms pointed to up to four antagonistic compounds, concerning the estrogenic and androgenic activity in two leaves and one fruit extracts, whereas no androgens were detected. From selected bioactive zones, syringin, vanilloloside, salidroside, α-hederin, cuneataside E, botulin, and oleanolic acid were tentatively assigned in the leaf extract using HPTLC–heart-cut–HPLC–HESI-HRMS, while they were salidroside and quinatic acid in the fruit extract. The *C. ternatea* flower extracts exhibited antioxidant and antibacterial properties, as well as the inhibition of AChE, BChE, tyrosinase, β-glucuronidase, and α-amylase. Only two extracts revealed estrogens in the multiplex pYAVES bioautograms, whereas no genotoxins and no androgens were detected. From the selected bioactive zones, taraxerol, kaempherol-3-rutinoside, kaempferol-3-glucoside, quercetin-3-rutinoside, and octadecenoic acid were tentatively assigned. Future studies could focus on the detailed mechanisms of action of the proposed compounds.

## Figures and Tables

**Table 1:** HPTLC–heart-cut–HPLC–HESI-HRMS signals obtained in the positive and negative ionization modes and tentative assignment of the four active compound zones **I**–**IV** in *A. quinata* leaf and fruit.

**Zone**	***hR*** **_F_**	**Formula**	**Calculated** **Mass [Da]**	**Observed** **Mass [Da]**	**Adduct** **Ions**	**Mass Error** **(ppm)**	**Tentative** **Assignment**
**I**	52	C_17_H_24_O_9_	372.1420	431.1563 395.1308	[M+CH_3_COO]^−^ [M+Na]^+^	−1.03 1.07	Syringin
		C_14_H_20_O_8_	316.1158	315.1082 339.1054	[M−H]^−^ [M+Na]^+^	1.03 −1.12	Vanilloloside
		C_14_H_20_O_7_	300.1209	299.1132 323.1097	[M−H]^−^ [M+Na]^+^	1.42 1.30	Salidroside
**II**	60	C_41_H_66_O_12_	750.4554	749.4489 773.4433	[M−H]^−^ [M+Na]^+^	−1.04 1.71	α-Hederin
		C_24_H_40_O_11_	504.2571	527.2451	[M+Na]^+^	2.32	Cuneataside E
**III**	65	C_30_H_50_O_2_	442.3811	465.3706	[M+Na]^+^	−0.60	Betulin
		C_30_H_48_O_3_	456.3604	479.3498	[M+Na]^+^	−0.37	Oleanolic acid
**IV**	55	C_14_H_20_O_7_	300.1209	299.1134 323.1095	[M−H]^−^ [M+Na]^+^	0.63 1.92	Salidroside
		C_29_H_44_O_4_	456.3240	455.3170 479.3138	[M−H]^−^ [M+Na]^+^	−0.61 −1.21	Quinatic acid

**Table 2:** HPTLC–heart-cut–HPLC–HESI-HRMS signals obtained in the positive and negative ionization modes and tentative assignment of the two active zones **I** and **II** in *C. ternatea* flowers.

**Zone**	***hR*_F_**	**Formula**	**Calculated** **Mass [Da]**	**Observed** **Mass [Da]**	**Adduct** **Ions**	**Mass Error** **(ppm)**	**Tentative** **Assignment**
**I**	46	C_30_H_50_O	426.3862	449.3751	[M+Na]^+^	0.69	Taraxerol
		C_27_H_30_O_15_	594.1585	593.1525 617.1483	[M−H]^−^ [M+Na]^+^	−2.13 −0.91	Kaempferol-3-rutinoside
		C_27_H_30_O_16_	610.1534	609.1473 633.1435	[M−H]^−^ [M+Na]^+^	−1.91 −1.34	Quercetin-3-rutinoside
**II**	56	C_18_H_34_O_2_	282.2569	281.2489 305.2453	[M−H]^−^ [M+Na]^+^	−0.95 −0.59	Octadecenoic acid
		C_21_H_20_O_11_	448.1006	447.0937 471.0902	[M−H]^−^ [M+Na]^+^	−0.89 −0.85	Kaempferol-3-glucoside

## Data Availability

Data are available upon reasonable request.

## Supplementary Materials

The following supporting information can be downloaded at: https://www.mdpi.com/article/10.3390/molecules28072893/s1, Figure S1: Selection of extraction solvent; Figure S2: HPTLC−pYAVAS bioautograms; Table S1: List of samples; Table S2: Mobile phase optimization.

## Sample Availability

Samples are available from the authors on request.
