# Supplementary material for: Effect-Directed Profiling of Akebia quinata and Clitoria ternatea via High-Performance Thin-Layer Chromatography, Planar Assays and High-Resolution Mass Spectrometry
Source: Molecules. 2023 Mar 23;28(7):2893. doi: 10.3390/molecules28072893 (PMC10096148; doi:10.3390/molecules28072893)
Supplement: Supplementary file 1 [file molecules-28-02893-s001.zip › molecules-2275916-supplementary.pdf]

Supplementary Material

Effect-Directed Profiling of *Akebia quinata* and *Clitoria ternatea* via High-Performance Thin-Layer Chromatography, Planar Assays and High-Resolution Mass Spectrometry

Hanna Nikolaichuk [1-3], Irena M. Choma [2], Gertrud E. Morlock [1,*]

[1] Chair of Food Science, Institute of Nutritional Science, Justus Liebig University Giessen, Heinrich-Buff-Ring 26-32, 35392 Giessen, Germany

[2] Department of Chromatography, Faculty of Chemistry, Maria Curie-Sklodowska University sq.3, 20031 Lublin, Poland

[3] Department of Bioanalytics, Faculty of Biomedicine, Medical University of Lublin, Jaczewskiego st. 8b, 20090, Lublin, Poland

*Correspondence: gertrud.morlock@uni-giessen.de

Table S1 List of the investigated *Akebia quinata* and *Clitoria ternatea* samples

ID	Plant part	Product name	Provider	Country of origin
***Akebia quinata* D.**				
A1	Leaf	-	Botanical garden, Giessen, Germany	Germany
A2	Leaf	-	Palmengarten, Frankfurt, Germany	Germany
A3	Leaf	-	Jagiellonian University, Cracow, Poland	Poland
A4	Fruit	-	Jagiellonian University, Cracow, Poland	Poland
***Clitoria ternatea* L.**				
C1	Flower	Butterfly pea tea	Redplum Private, Faridabad, India	India
C2	Flower	Butterfly pea flower (Klitorienblüte)	Herbathek, Berlin, Germany	Sri Lanka
C3	Flower	Niebieska *Clitoria ternatea*	Proherbis, Dębowiec, Poland	China
C4	Flower	Kwiat klitorii ternateanskiej	Dary Natury, Grodzisk, Poland	Poland

Table S2 Mobile phase optimization for *Akebia quinata* (4 µL/band) and *Clitoria ternatea* (5 µL/band) extracts on HPTLC plate silica gel 60 F_{254} separated with the respective mobile phase system

Akebia quinata			
MP	Solvent composition	Ratio (*V/V*)	Chromatograms at UV 254 nm, FLD 366 nm and bioluminescence after *Aliivibrio fischeri* bioassay
1	Ethyl acetate-methanol-water	77:13:10	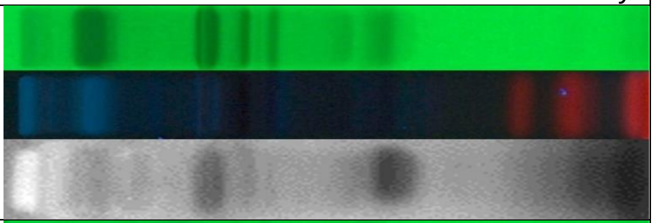
2	Ethyl acetate-methanol-water-acetic acid	77:13:10:1	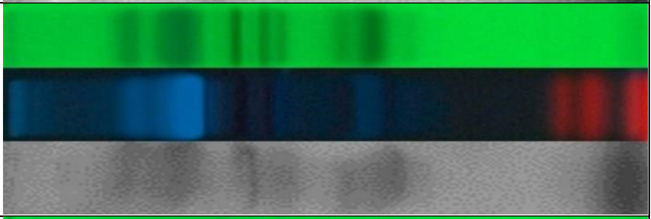
3	Ethyl acetate-methanol-water-acetic acid	77:13:10:2	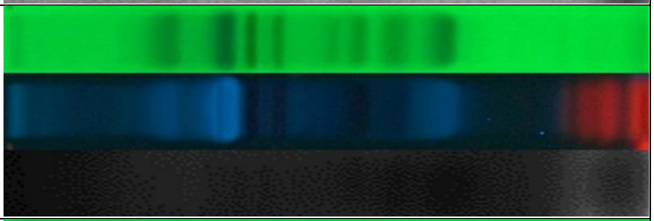
4	Ethyl acetate-acetonitrile-water	7:2:1	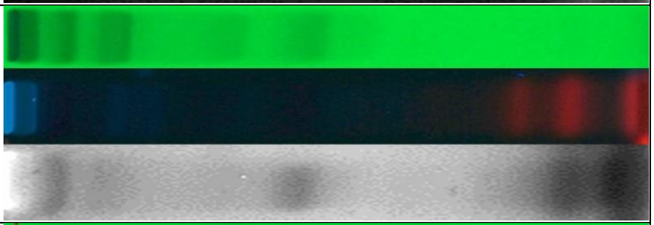
5	Ethyl acetate-acetonitrile-water	5:4:1	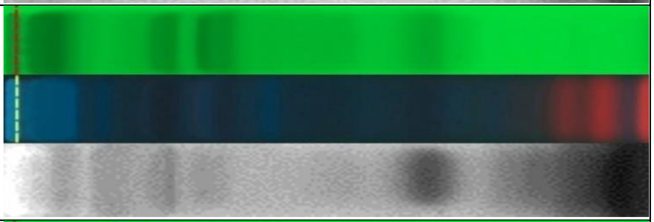
6	Ethyl acetate-methanol-water-acetic acid	77:13:10:1.5	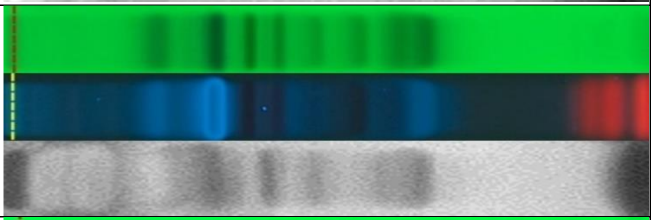
7	Ethyl acetate-methanol-water-acetic acid	70:15:10:2	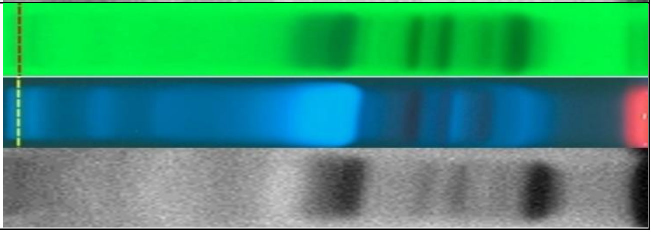

8	Ethyl acetate-acetonitrile-water-acetic acid	50:40:10:1	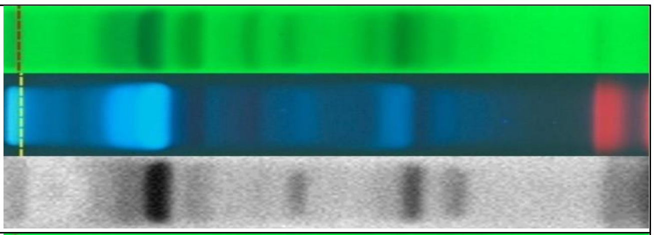
9	Ethyl acetate-methanol-water-acetic acid	70:15:10:1	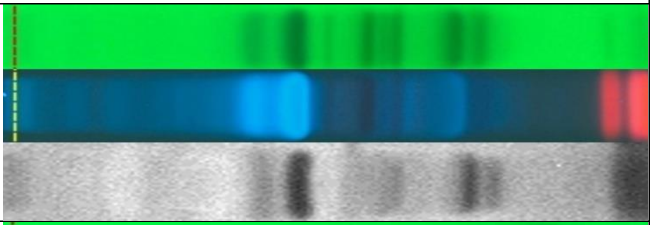
10	Ethyl acetate-methanol-water-acetic acid	50:40:10:1	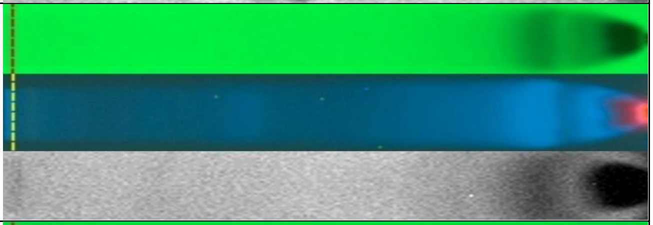
11	Ethyl acetate-methanol-water-acetic acid	60:30:10:1	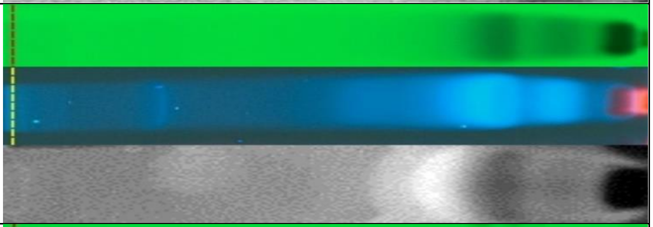
12	Ethyl acetate-methanol-water-acetic acid	70:20:10:1	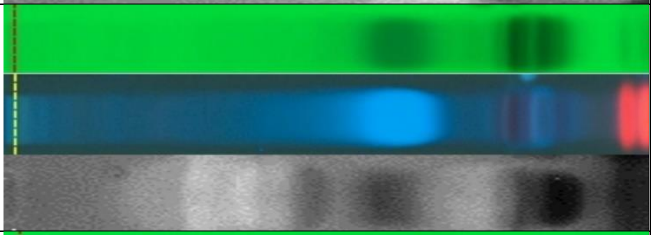
13	Ethyl acetate-acetonitrile-water-acetic acid	40:50:10:1	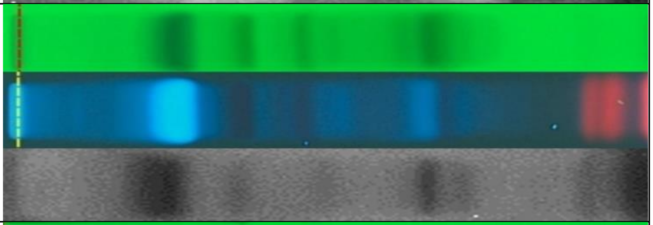
14	Focusing (methanol),<br>3 cm<br>Ethyl acetate-water	10:1	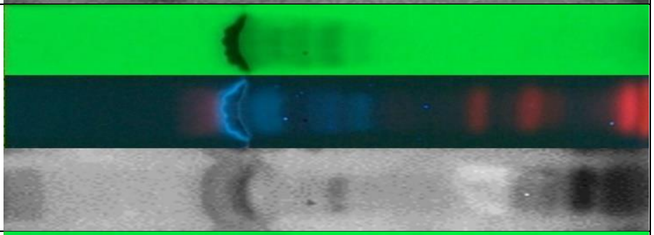
15	Focusing (methanol,<br>2 x acetone),<br>2 cm<br>Ethyl acetate-methanol-water-acetic acid	70:15:15:1	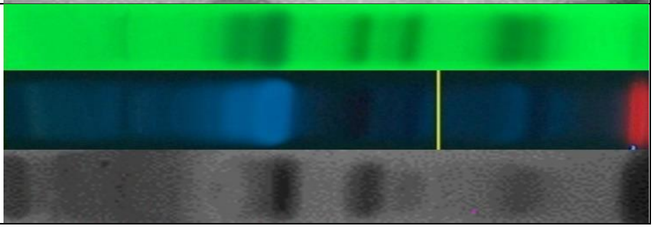

16	Ethyl acetate-methanol-water-acetic acid	70:15:15:1	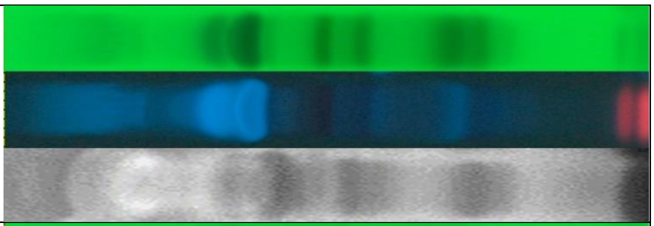
17	Focusing (2 x acetone), 2 cm<br>Ethyl acetate-methanol-water-acetic acid	7:1.5:1.5:0.1	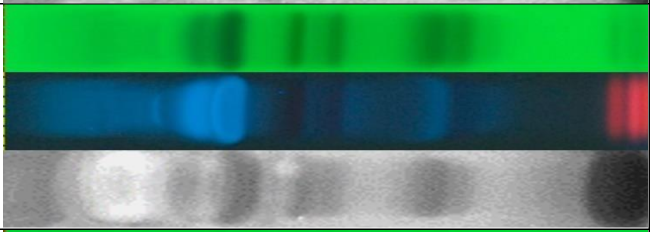
16	Ethyl acetate-methanol-water- acetic acid	7:1.5:1.5:0.1	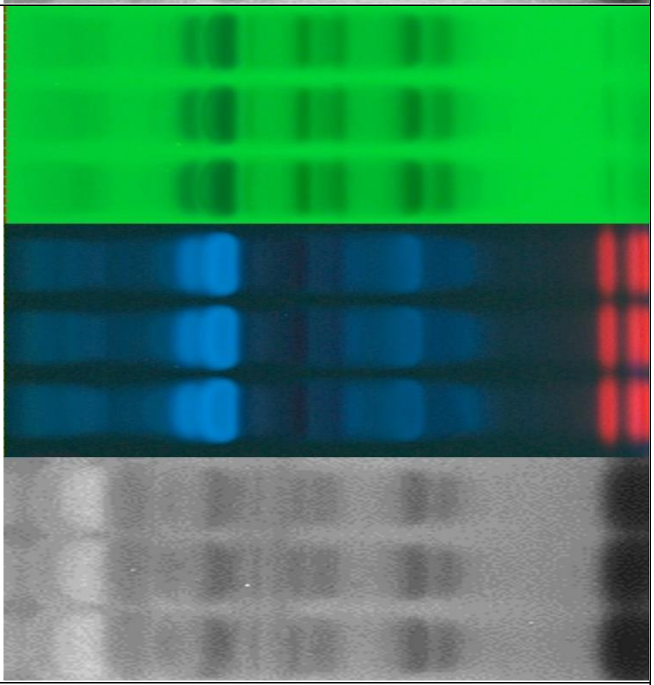
Clitoria ternatea			
MP	Solvent composition	Ratio (*V/V*)	Chromatograms at UV 254 nm, FLD 366 nm and bioluminescence after *Aliivibrio fischeri* bioassay
1	Ethyl acetate-methanol-water	77:13:10	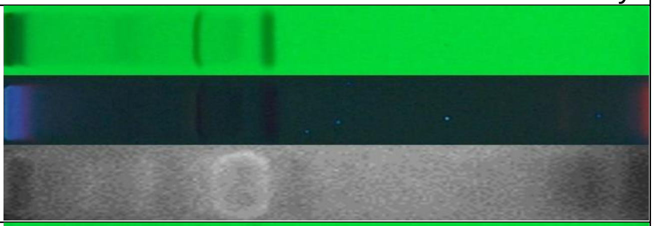
2	Ethyl acetate-methanol-water-acetic acid	77:13:10:1	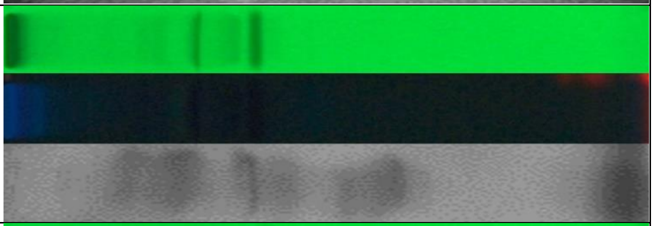
3	Ethyl acetate-methanol-water-acetic acid	77:13:10:2	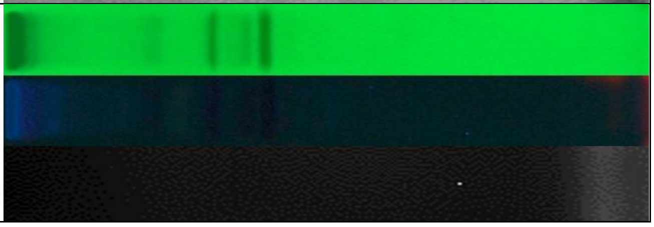

4	Ethyl acetate-acetonitrile-water	7:2:1	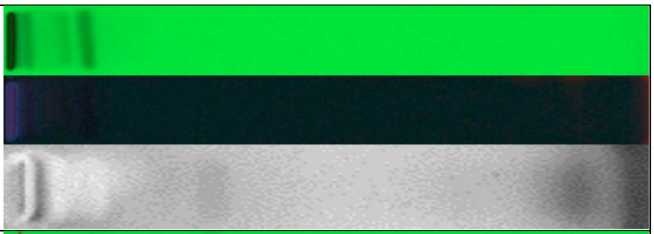
5	Ethyl acetate-acetonitrile-water	5:4:1	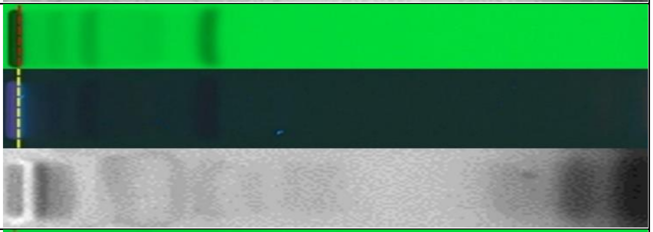
6	Ethyl acetate-methanol-water-acetic acid	77:13:10:1.5	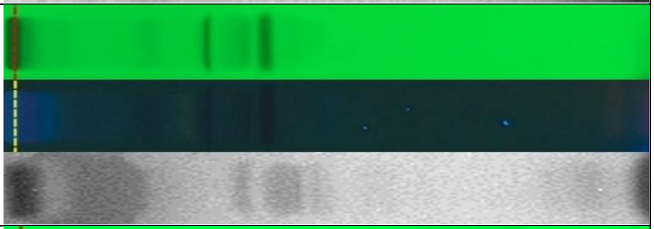
7	Ethyl acetate-methanol-water-acetic acid	70:15:10:2	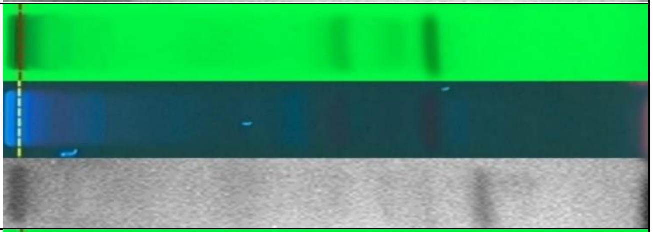
8	Ethyl acetate-acetonitrile-water-acetic acid	50:40:10:1	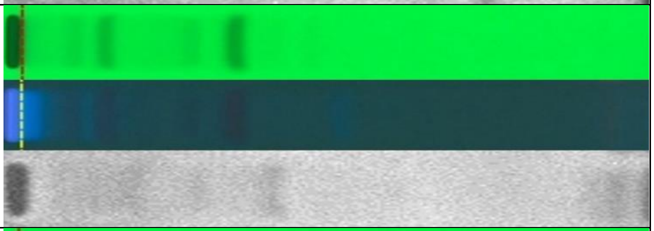
9	Ethyl acetate-methanol-water-acetic acid	70:15:10:1	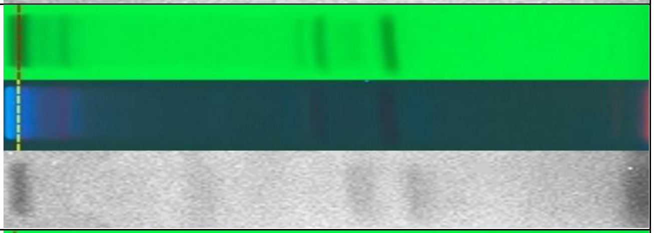
10	Ethyl acetate-methanol-water-acetic acid	50:40:10:1	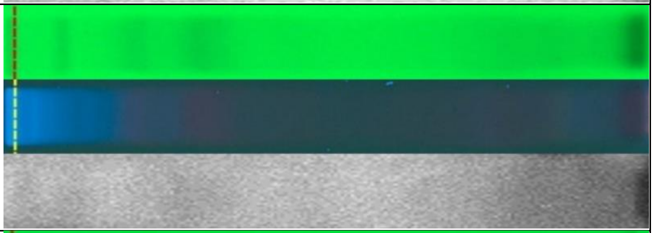
11	Ethyl acetate-methanol-water-acetic acid	60:30:10:1	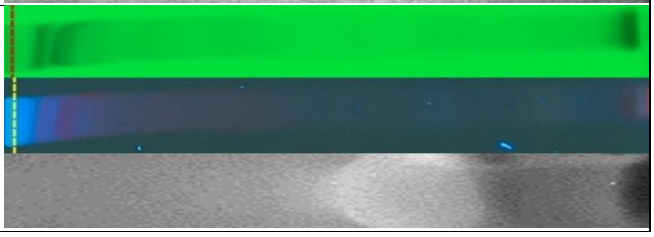

12	Ethyl acetate-methanol-water-acetic acid	70:20:10:1	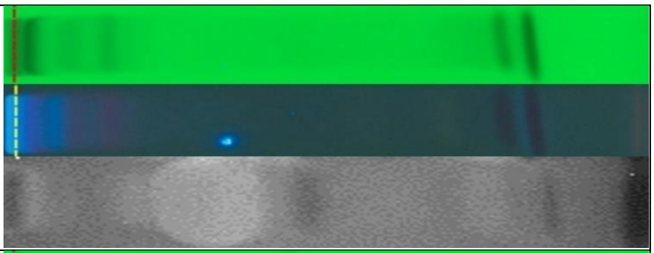
13	Ethyl acetate-acetonitrile-water-acetic acid	40:50:10:1	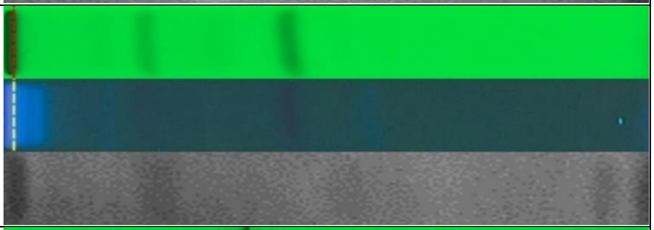
14	Focusing (methanol),<br>3 cm<br>Ethyl acetate-water	10:1	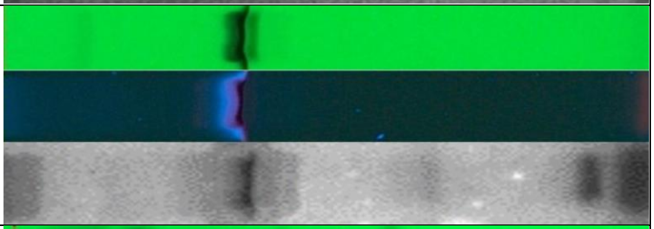
15	Focusing (methanol, 2 x acetone),<br>2 cm<br>Ethyl acetate-methanol-water-acetic acid	70:15:15:1	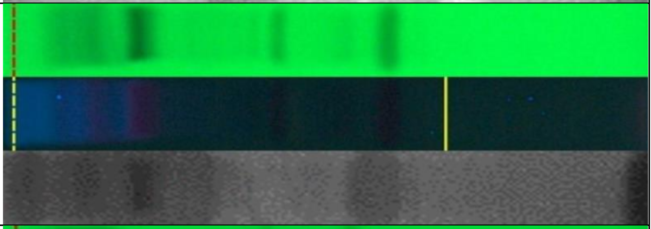
16	Ethyl acetate-methanol-water-acetic acid	70:15:15:1	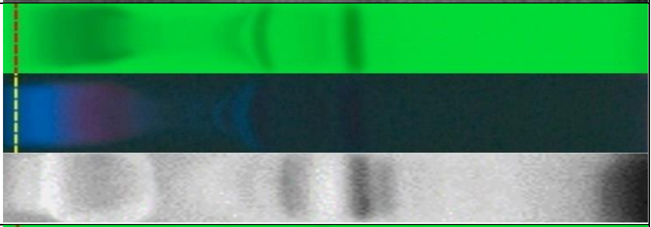
17	Focusing (2 x acetone),<br>2 cm<br>Ethyl acetate-methanol-water-acetic acid	7:1.5:1.5:0.1	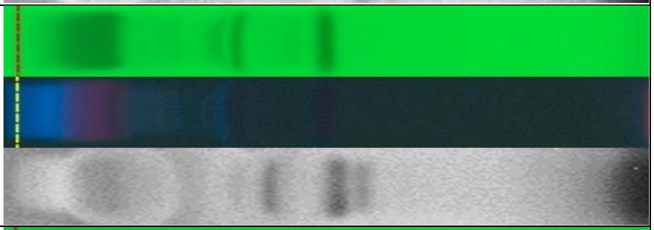
16	Ethyl acetate-methanol-water- acetic acid<br><br>3, 3,5 and 4 µL/band	7:1.5:1.5:0.1	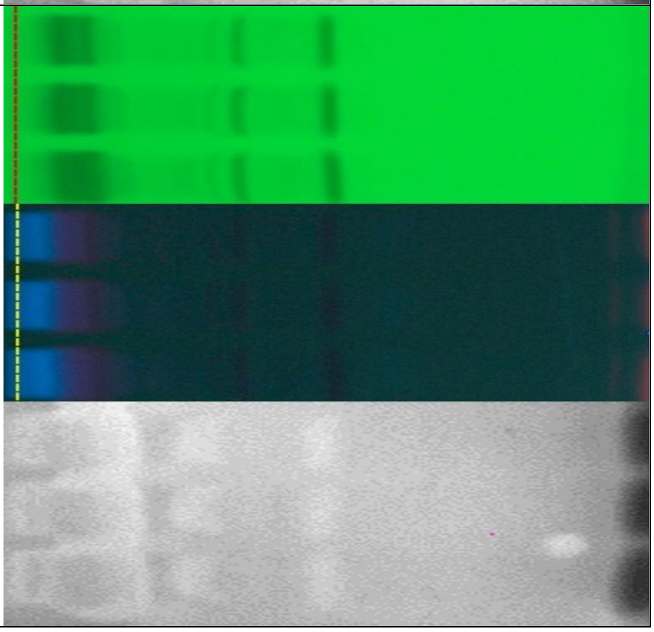

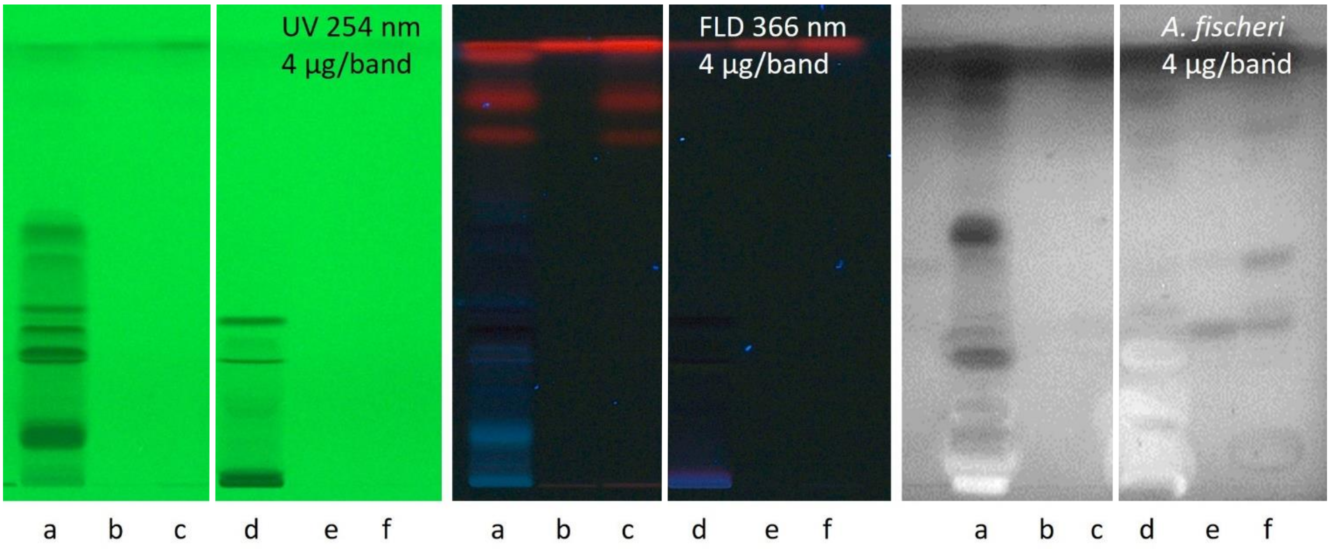

Figure S1. Selection of extractant for *Akebia quinata* (a–c) and *Clitoria ternatea* (d–f): HPTLC chromatograms at UV 254 nm and FLD 366 nm as well as *A. fischeri* bioautogram (bioluminescence depicted as greyscale image) showing extraction either with (a/d) methanol–water 4:1 or (b/e) *n*-hexane or (c/f) ethyl acetate, all separated on the HPTLC plate silica gel 60 F_{254} with ethyl acetate–methanol–water 7.7:1.3:1, *V*/*V*/*V*.

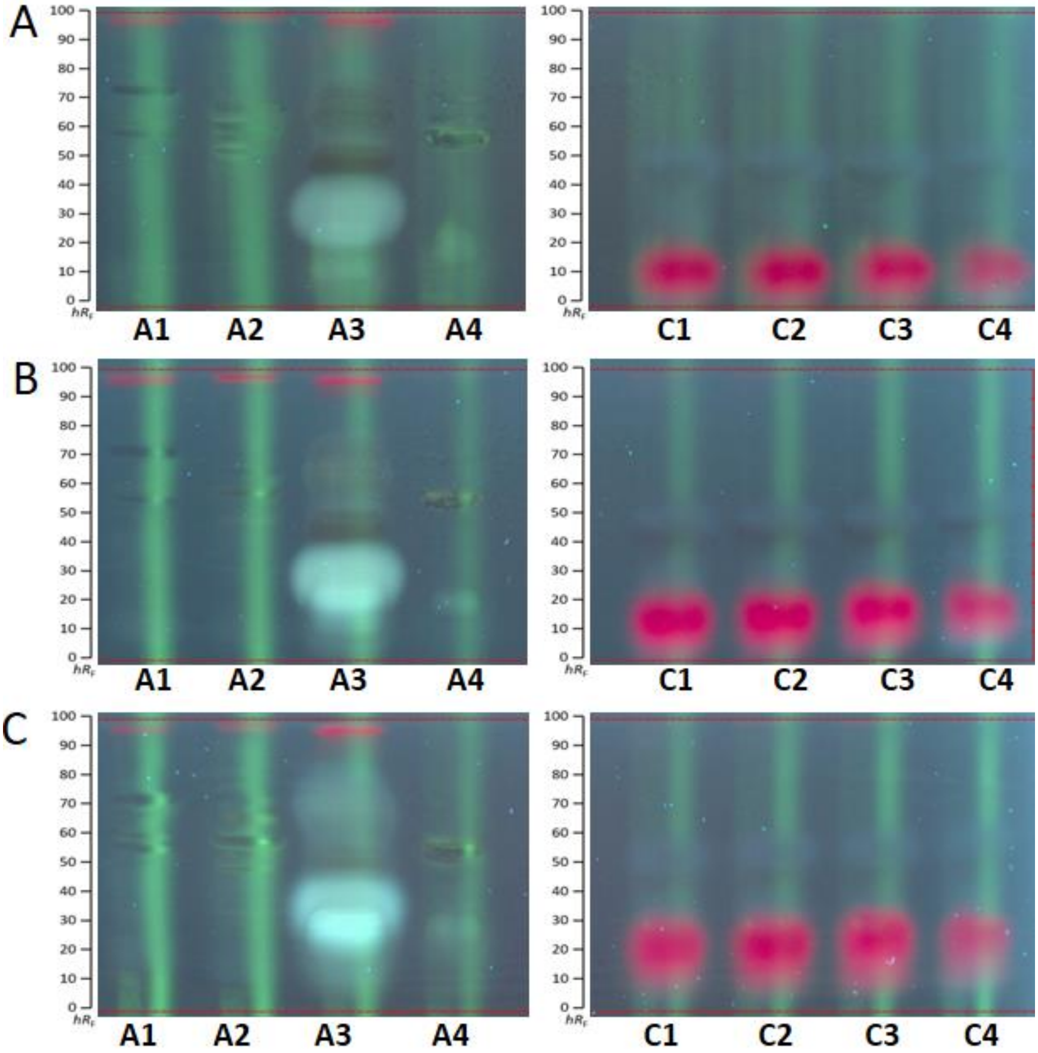

Figure S2. HPTLC–pYAVAS bioautograms at FLD 254 nm of *Akebia quinata* (A1–A4) and *Clitoria ternatea* (C1–C4) extracts (12 mm band, 10 µL/band, 1 mg/band each) on HPTLC plate neutralized with (A) sodium bicarbonate buffer (pH 7), (B) sodium acetate buffer (pH 7) and (C) citrate buffer (pH 12) separated with ethyl acetate–methanol–water–acetic acid 70:15:15:1, *V/V/V/V*.
